# Mr. Armstrong, on Hydrophobia

**Published:** 1808-10

**Authors:** Charles Armstrong

**Affiliations:** London


### *To the Editors of the Medical and Physical Journal.*

Gentlemen,

In the last number of the Medical and Physical Journal, Dr. Beddoes and Mr. Smith, have related two cases of Hydrophobia; in one of which, caustic was applied to the bitten part the day following the injury; in the other, two days after; and in both without success. Discouraging as the event of these cases has been, I should not hesitate, in similar circumstances, where excision had been refused or neglected, or where it could not be employed with safety, to recommend the most speedy removal of the wounded part by an active caustic.

Every instance of preservation from Hydrophobia, after the bite of a rabid animal, that is made public, must tend to promote the means of preservation; and, where those means have been successful, after a delay of two days from the time the injury was received, the promulgation of the event must give confidence and encouragement to medical men, and induce them to effect the destruction of the bitten part, as the only prophylactic on which they can rely with any degree of confidence. That it does not always afford perfect security, the before-mentioned cases will prove; but, that it may sometimes be so managed, as to preserve life, one of the following instances will attest.

Master Hill, aged ten years, was bit by a dog on the external part of the left heel, on the evening of the 21st of June, 1807. The teeth of the animal had penetrated the cutis in two places, which were immediately washed with brandy, and had a compress of linen wet with the same spirit bound over them. His father gave the following account of the accident. The boy had been accustomed to teize and provoke the dog, which was a fierce creature, and had snapped at him once or twice before. On this occasion, the dog was following a proud bitch, and on the boy coming in his way, he seized him by the heel, and was with some difficulty disengaged from his hold. The dog had never refused food, nor exhibited any symptom of indisposition, madness, therefore, was not suspected, though several mad dogs had been seen in the neighbourhood, and this dog was daily in the streets.

On the morning of the 23d, the dog flew at the maid servant, and bit her on the second finger of the right hand, and in two places on the back of the hand. He had also this morning, torn that bitch he was following when he bit the boy, and likewise two cats, and evinced a strong inclination to bite every thing in his way. The maid came directly to my house, and brought the boy with her. The wounds on her hand and finger, though slight, bled freely. There was now in my mind little doubt of the rabid state of the animal, I therefore urged the immediate removal of the bitten parts, to which the woman consented, and they were carefully cut out. After suffering the wounds to bleed for ten minutes, they were washed with alcohol, and dressed with emplastrum cantharidis spread on dossils of lint. The same treatment was proposed for the boy; but as the father would not consent to excision, or to any other means of prevention till the evening, a caustic of kali purum and soft soap was then applied over the bitten parts, and suffered to remain on all night. This was at least forty-eight hours after the bite. The caustics made deep sloughs on the boy's heel, and as soon as the eschars began to separate, the edges of the ulcers were dressed daily with emplastrum cantharidis on lint.

June 25th. The dog having been alternately howling, and furious, biting and tearing every thing in his way, was destroyed.

July 2d. One of the cats that were bitten on the 23d of last month, seized a girl of eleven years of age, by the leg, leg, a little above the ankle, and bit her in four places. The father obstinately refusing to allow the wounded parts to be removed by the knife, they were carefully washed with salt and water, and the caustic of kali purum and soft soap applied to each. The ulcers were dressed as in the other cases.

It is here necessary to observe, that one of the cats died the day she was bitten, of the immediate effect of the injury. The other cat, and the bitch, were shut up, but they got out by the most culpable neglect and inattention. After the mischief was done, the cat was killed, and the bitch secured with a chain.

July 4th. The bitch had a wild and eager aspect, with redness about her eyes. The following day she refused food. On the 6th, her appearance was haggard and miserable, the face of the animal exhibiting a striking and peculiar expression of wretchedness, accompanied with a discharge of ropy saliva from the corners of the mouth.—The next morning she died.

In these three instances a copious discharge was kept up from the ulcers, by the daily application of blistering plaster, for the space of a month, after which they were suffered to heel. During the whole process, the patients took from fifteen to thirty drops of aqua ammoniæ, twice a day, in two or three spoonfuls of water, and, I have the pleasure to add, are now in perfect health.

If it be allowed from this relation, that the dog was rabid at the time he bit the boy, which, I presume, is too clearly authenticated by the subsequent events to admit of doubt, it will follow, that the fact of preservation, after a delay of two days, is evidently made out; and, consequently, that it is our duty where excision has not been performed, to apply an active caustic at any period, and also to promote a free discharge by the daily use of a stimulating application to the ulcerated surface, for at least four weeks.

In the eighteenth volume of your Journal, page 550, is related a melancholy case of Hydrophobia, by Dr. Moseley, in which there does not appear to have been any attempt made to avert the fatal issue; but in your seventeenth volume, page 271, Mr. Hicks details a case, at some length, of recovery after the accession of hydrobic symptoms. This case, however, has been controverted by some gentlemen, who have contended that it was not an instance of genuine hydrophobia. Mr. Hicks, on the other hand, supports his opinion with considerable ability.

The case may be doubtful; but if it was hydrophobia, we must surely attribute the cure to the caustic, and not to the fœtid mixture.

I am, &c.

CHARLES ARMSTRONG.

*London, Sept.* 10, 1808.

